# Exploring the causal relationship between gut microbiota and multiple myeloma risk based on Mendelian randomization and biological annotation

**DOI:** 10.3389/fmicb.2024.1310444

**Published:** 2024-02-12

**Authors:** Zuxi Feng, Minjing Liao, Jun Bai, Yanhong Li, Yue Chen, Li Zhang, Xuege Guo, Lijuan Li, Liansheng Zhang

**Affiliations:** ^1^Department of Hematology, Lanzhou University Second Hospital, Lanzhou, China; ^2^Second Clinical Medical College, Lanzhou University, Lanzhou, China; ^3^Key Laboratory of the Hematology of Gansu Province, Lanzhou University Second Hospital, Lanzhou University, Lanzhou, China

**Keywords:** multiple myeloma, gut microbiota, Mendelian randomization, biological annotation, immunoregulation

## Abstract

**Introduction:**

The microbial genome-wide association studies (mbGWAS) have highlighted significant host-microbiome interactions based on microbiome heritability. However, establishing causal relationships between particular microbiota and multiple myeloma (MM) remains challenging due to limited sample sizes.

**Methods:**

Gut microbiota data from a GWAS with 18,340 participants and MM summary statistics from 456,348 individuals. The inverse variance-weighted (IVW) method was used as the main bidirectional Mendelian randomization (MR) analysis. To assess the robustness of our results, we further performed supplementary analyses, including MR pleiotropy residual sum and outlier (MR-PRESSO) test, MR-Egger, Weighted median, Simple mode, and Weighted mode. Moreover, a backward MR analysis was conducted to investigate the potential for reverse causation. Finally, gene and gene-set-based analyses were then conducted to explore the common biological factors connecting gut microbiota and MM.

**Results:**

We discovered that 10 gut microbial taxa were causally related to MM risk. Among them, family *Acidaminococcaceae*, *Bacteroidales* family S24-7, family *Porphyromonadaceae*, genus *Eubacterium ruminantium group*, genus *Parabacteroides*, and genus *Turicibacter* were positively correlated with MM. Conversely, class *Verrucomicrobia*, family *Verrucomicrobiaceae*, genus *Akkermansia*, and order *Verrucomicrobiales* were negatively correlated with MM. The heterogeneity test revealed no Heterogeneity. MR-Egger and MR-PRESSO tests showed no significant horizontal pleiotropy. Importantly, leave-one-out analysis confirmed the robustness of MR results. In the backward MR analysis, no statistically significant associations were discovered between MM and 10 gut microbiota taxa. Lastly, we identified novel host-microbiome shared genes (AUTS2, CDK2, ERBB3, IKZF4, PMEL, SUOX, and RAB5B) that are associated with immunoregulation and prognosis in MM through biological annotation.

**Discussion:**

Overall, this study provides evidence supporting a potential causal relationship between gut microbiota and MM risk, while also revealing novel host-microbiome shared genes relevant to MM immunoregulation and clinical prognosis.

## 1 Introduction

Multiple myeloma (MM) is a clonal plasma cell malignant tumor originating in the bone marrow, characterized by the excessive production of monoclonal immunoglobulins (Joshua et al., 2019). Its global incidence has steadily increased, making it the second most prevalent blood cancer after leukemia. Importantly, MM is not a single entity but rather a heterogeneous disease (Owens, 2020). Clinical presentations vary from asymptomatic cases to life-threatening conditions, with common manifestations including fatigue, bone pain, hypercalcemia with renal insufficiency, anemia, and hyperproteinemia (Angtuaco et al., 2004). Diagnosis relies on multidisciplinary approaches encompassing orthopedics, radiology, nuclear medicine, hematology, and oncology (Caers et al., 2018). In recent decades, patient survival rates have significantly increased due to the implementation of proteasome inhibitors and immunomodulatory drugs. However, these treatments are not curative, and MM often recurs, necessitating further intervention (Laubach et al., 2015). Consequently, the quest for optimized treatment strategies for MM remains a significant long-term challenge.

The human intestine hosts a diverse and intricate microbial community known as gut microbiota, which derives from various sources, including dietary intake and probiotic supplements. These microbiotas are essential for maintaining mucosal barrier integrity and offering numerous other health benefits (Badgeley et al., 2021). Currently, gut microbiota is recognized as both a contributor to and a safeguard against various human diseases, including its intricate involvement in the pathogenesis of MM (Park et al., 2022). Conversely, cancer can influence the host’s gut microbiota, potentially leading to microbiota disruption and tumor progression (Yonekura et al., 2022). The gut microbiota within a human host represents a partially heritable phenotype, and correlations exist between the host’s genotype and gut microbiota variations (Goodrich et al., 2014). The interactions between hosts and microorganisms are intricate, with distinct mechanisms governing gut microbiota’s impact on host health and disease. Current scientific research is rapidly advancing toward enhancing cancer treatment strategies by assessing human microbial composition and function and subsequently implementing targeted regulation (Park et al., 2022). Although limited sample sizes have hindered research in this area, resulting in limited published mbGWAS studies, mbGWAS have still revealed significant insights (Lopera-Maya et al., 2022). Over the past 5 years, research into the relationship between gut microbiota and MM has steadily progressed. The latest research reveals that in MM patients, the gut microbiome’s enrichment of *Citrobacter freundii* induces drug resistance by increasing ammonium levels through the transmembrane channel protein SLC12A2 (Zhu et al., 2024). Simultaneously, a potential therapeutic strategy is proposed, utilizing furosemide sodium to inhibit ammonium uptake and enhance treatment efficacy. Gut microbiota diversity in autologous hematopoietic cell transplant recipients is initially reduced, resembling allogeneic transplants (Khan et al., 2021). Higher diversity during transplantation is associated with a lower risk of progression or death, highlighting a potential connection between gut microbiota and patient outcomes. Gut microbiota alterations and changes in short-chain fatty acid levels are associated with the progression of multiple myeloma, indicating potential therapeutic targets and predictors of treatment response (Rodriguez-Garcia et al., 2023). Thus, understanding the role of gut microbiota in MM is crucial, as investigating the causal relationship between gut microbiota and MM holds significant implications for advancing our knowledge of disease mechanisms and potential therapeutic interventions.

In recent years, the role of immunology in tumor pathogenesis has gained widespread attention, introducing a new era in cancer treatment through immunotherapy. Medical research has elucidated the vital role of microbiota in regulating tumor immune surveillance and participating in cancer pathogenesis and progression (Liu et al., 2021). The latest findings demonstrate that fiber-rich diets and fermented foods can modulate microbial composition, activity, and host immune status (Wastyk et al., 2021). Consequently, gut microbiota-targeted immunotherapy has recently surfaced as an innovative approach in treating cancer (Zhou et al., 2021). Calcinotto et al. (2018) discovered that the gut microbiota plays a role in the progression of MM by affecting the differentiation and migration of Th17 cells in the bone marrow. Furthermore, an immunological connection between the gut and the transition from asymptomatic to symptomatic MM has been confirmed (Calcinotto et al., 2018). In MM patients, the gut microbiome exerts influence not only on disease progression but also on treatment response and treatment-related toxicity (Ahmed et al., 2020). Recent research highlights microbiota’s role in promoting inflammation and influencing MM’s disease development, including transplantation (Pianko and Golob, 2022).

Despite the challenges of obtaining real-time microbiome data in clinical practice, retrospective studies have started illuminating the relationship between changes in particular microbial characteristic and MM (Zhang et al., 2022). Due to confounding factors such as lifestyle, environment, host gene mutations, and potential reverse causality, the precise association between genes in MM patients and gut microbiota remains underexplored. Mendelian Randomization (MR) using two-sample analysis is a robust approach that leverages inherited genetic variations as instrumental variables (IVs) to investigate causal relationships between exposure and outcome traits. MR leverages large GWAS data to study the associations between risk factors and diseases while effectively controlling non-genetic confounding variables, leading to more precise and reliable results. Currently, MR is extensively utilized for investigating the causal link between gut microbiota and diseases (Shi et al., 2023; Zeng Y. et al., 2023). Although current research results suggest a potential interaction between gut microbiota and MM, there is still considerable research potential. Thus, we aim to delve deeper and comprehensively explore the causal relationship between gut microbiota and MM through MR analysis. Identifying therapeutic targets, promoting precision medicine, and uncovering prognostic indicators are crucial aspects in the diagnosis and treatment of MM.

In our study, we utilized bidirectional two-sample MR analyses to investigate potential causal links between microbial characteristics and MM. Furthermore, we offered a biological annotation for the noteworthy connections between gut microbiota and MM.

## 2 Materials and methods

### 2.1 Study design

**FIGURE 1:**
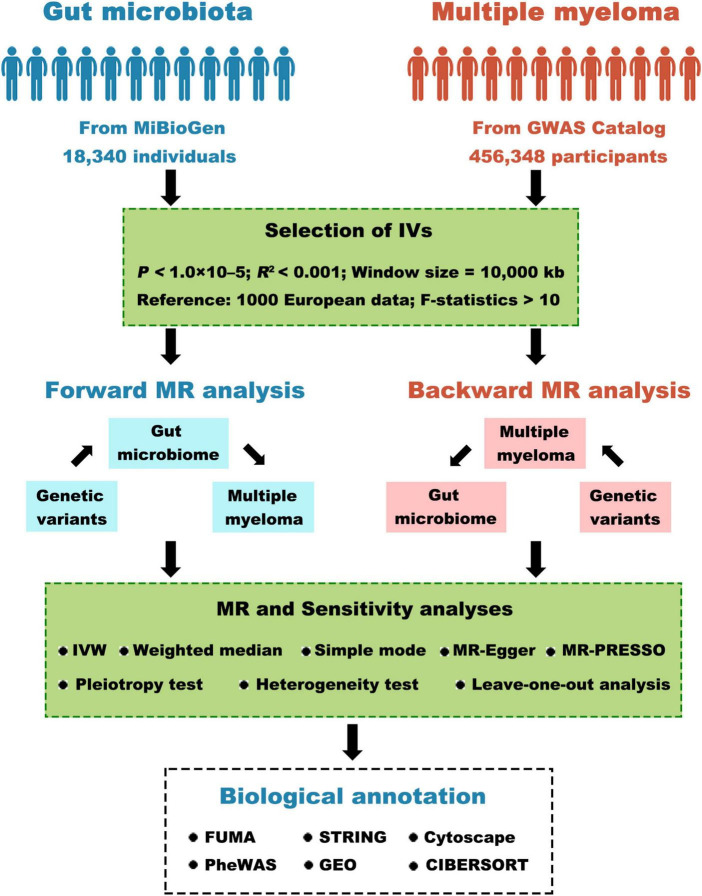
Visual depiction of the research study. GWAS, genome-wide association study; IV, instrumental variables; MR, Mendelian randomization; IVW, inverse-variance weighted; MR-PRESSO, MR pleiotropy residual sum and outlier; GEO, gene expression omnibus.

Figure 1 depicts the comprehensive study design. In summary, our study involved extracting genetic variants linked to the respective exposure by analyzing GWAS summary statistics. We meticulously selected instrumental variables based on stringent criteria. We then conducted a bidirectional two-sample MR analysis using five distinct MR methods in a sequential manner. Next, a comprehensive series of sensitivity analyses, comprising the examination of heterogeneity, pleiotropy, and the leave-one-out test, were conducted to scrutinize the robustness of statistically significant associations. Lastly, we explored positional mapped genes and gene-set-based analyses using biological annotation.

### 2.2 Data sources

In this study, we acquired summary statistics for human MM from a previously published GWAS.^1^ This GWAS incorporated data from the UK Biobank and encompassed 456,348 individuals with European ancestry (Jiang et al., 2021). This dataset includes 564 cases of European ancestry and 455,784 controls of European ancestry. Additionally, summary statistics concerning the human gut microbiome in this study were derived from a GWAS involving 18,340 participants.^2^ Our study encompasses 24 cohorts, samples from single-ancestry cohorts include European (16 cohorts, *N* = 13,266), Middle-Eastern (1 cohort, *N* = 481), East Asian (1 cohort, *N* = 811), American Hispanic/Latin (1 cohort, *N* = 1,097), and African American (1 cohort, *N* = 114). Additionally, four cohorts were multi-ancestry (*N* = 2,571). The gene expression data for 138 samples were downloaded from the Gene Expression Omnibus (GEO)^3^ database with accession number GSE13591. To ascertain the relationship between the expression of hub genes and the overall survival of patients with MM, we utilized the Kaplan-Meier plotter database.^4^ Note: The acquisition dates for databases used in this study are as follows: MM GWAS data (August 20, 2023), gut microbiome data (September 7, 2023), GEO data (September 10, 2023), and Kaplan-Meier plotter database (September 12, 2023).

### 2.3 Instrumental variable (IV)

At first, we omitted 15 microbial characteristics that did not have specific names, resulting in 196 microbial features. Later, we chose IVs using a less strict significance threshold of *p* < 1.0 × 10^–5^ (Liu et al., 2023; Zeng C. et al., 2023), which was determined to be the best *p*-value threshold for choosing genetic predictors that account for a greater amount of variance in the outcome. Additionally, we employed several alternative thresholds 5.0 × 10^–6^ (He et al., 2022; Nolde et al., 2022) and 5.0 × 10^–8^ (Papiol et al., 2021; Yuan and Larsson, 2022) to choose IVs for gut microbial (Supplementary Table 1). To prevent biased results stemming from linkage disequilibrium (LD), we performed clumping analysis on European samples from the 1000 Genomes project using the TwoSampleMR package, with stringent criteria (*R*^2^ < 0.001 and a window size of 10,000 kb). With an *R*^2^ value of less than 0.001, we retained only the single nucleotide polymorphism (SNP) possessing the lowest *p*-value. Additionally, we excluded SNPs not found in the LD reference panel. To gauge the strength of the IVs, we computed F-statistics using the provided equation *R*^2^ = 2 × MAF × (1-MAF) × β^2^, *F* = *R*^2^ × (N–1–k)/ k (1–*R*^2^), where *R*^2^ represents the variance explained by the IVs, “N” denotes the data sample size, and “k” represents the count of SNPs included in the instrument (Bowden et al., 2016). Ultimately, we opted for independent SNPs linked to MM as IVs, employing a threshold of 1.0 × 10^–5^ (Supplementary Table 2), which we utilized in subsequent MR analyses. In the backward MR analysis, we chose IVs linked to MM at various cutoff levels (1.0 × 10^–5^, 5.0 × 10^–6^, and 5.0 × 10^–8^) (Supplementary Table 3). In contrast to the forward analyses, the results at a threshold of 5.0 × 10^–8^ as the discovery set, while treating the other thresholds as validation criteria.

### 2.4 Statistical analysis

In this study, we employed various approaches to investigate potential causal connections between gut microbiota and MM, such as MR-PRESSO, IVW, weighted median, MR-Egger regression, simple mode, and weighted model. Our primary MR analysis relied on the IVW method (Yavorska and Burgess, 2017). The IVW method integrates the Delta method and Wald estimates for every SNP, producing a holistic assessment of the gut microbiota’s influence on MM (Song et al., 2023). Without horizontal pleiotropy, IVW results stay unbiased. We assessed the presence of overall horizontal pleiotropy by measuring genetic variant heterogeneity using the Q statistic and examining the intercept in the MR-Egger test to validate MR assumptions (Bowden et al., 2015). To address horizontal pleiotropy, we conducted sensitivity assessments employing simple mode, weighted median, and weighted mode. We also used the MR-PRESSO approach to conduct global and outlier tests in order to detect potential outliers. Subsequently, we obtained corrected association results by excluding these potential outliers (Verbanck et al., 2018). To assess the potential prevalence of particular variants in the process of estimating causality, we conducted leave-one-out examinations. This involved systematically omitting each SNP from the evaluation and subsequently reassessing the causative connection (Corbin et al., 2016).

Additionally, we explored the potential impact of directional pleiotropy by investigating the secondary phenotypes associated with each of the SNPs utilized as instrumental variables. This analysis was conducted using the PhenoScanner database, and the data was accessed on August 30, 2023, via the following link: http://www.phenoscanner.medschl.cam.ac.uk/. After confirming the connection between microbiota features and MM through MR methods, we proceeded with backward MR analyses. In this analysis, we examined MM as the exposure variable and assessed microbial features as the outcomes. “Mendelian Randomization,” “TwoSampleMR,” and “MRPRESSO” packages in the open-source statistical software R (version 4.2.3) were employed for our MR analyses. The connections between the human gut microbiota and MM risk were quantified using odds ratios (ORs) accompanied by 95% confidence intervals (CIs). Statistical significance was confirmed when two-sided *p*-values were below 0.05.

### 2.5 Biological annotation

Initially, we annotated the genes mapped to instrumental variants’ positions between gut microbiota and MM utilizing the GWAS catalog provided with FUMA (Welter et al., 2014). We then employed integrated hypergeometric tests within the FUMA platform, further enhancing our understanding of the previously reported associations between mapped genes and various phenotypes (Watanabe et al., 2017). Subsequently, we constructed protein–protein interaction (PPI) networks using genes mapped to instrumental variants for both MM and our MR analysis results for mbGWAS. We utilized STRING version 12.0^5^ and Cytoscape (version 3.8.2) to obtain and visualize the connected PPI network. We have determined the top ten hub genes through the Maximal Clique Centrality (MCC) approach, given that genes with numerous interconnecting links are pivotal for maintaining network stability (Xiao et al., 2023). Furthermore, we examined the gene expression profiles of central genes within bulk tissue through the FUMA portal. We assessed the impact of these hub genes on a wide range of characteristics by conducting phenome-wide association studies (PheWAS). We explored their pleiotropic effects in summary data for 4,756 complex traits and illnesses across 28 different domains, utilizing the GWAS ATLAS (Watanabe et al., 2019). To perform Gene Ontology (GO) and Kyoto Encyclopedia of Genes and Genomes (KEGG) pathway analysis for the hub genes, we used the “ClusterProfiler” and “org.Hs.eg.db” package in R software (version 4.2.3). In this study, we employed both *p*-value = 0.05 and *q*-value = 0.05 as filtering criteria for the analysis of GO terms and KEGG pathways. Additionally, we utilized the Kaplan-Meier plotter database to establish the association between hub gene expression and the prognosis of MM patients. Finally, we retrieved the expression data of hub genes from the GEO database to investigate their immune regulatory functions in MM.

## 3 Results

### 3.1 Forward MR analyses

**FIGURE 2:**
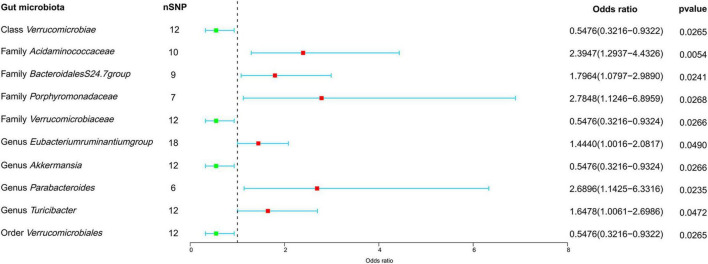
Forest plot depicting the forward MR findings. This forest plot illustrates potential causal connections between microbial characteristics and MM. The information is displayed in the form of odds ratios (OR) accompanied by their respective 95% confidence intervals (CI). nSNP, number of single nucleotide polymorphism.

**FIGURE 3:**
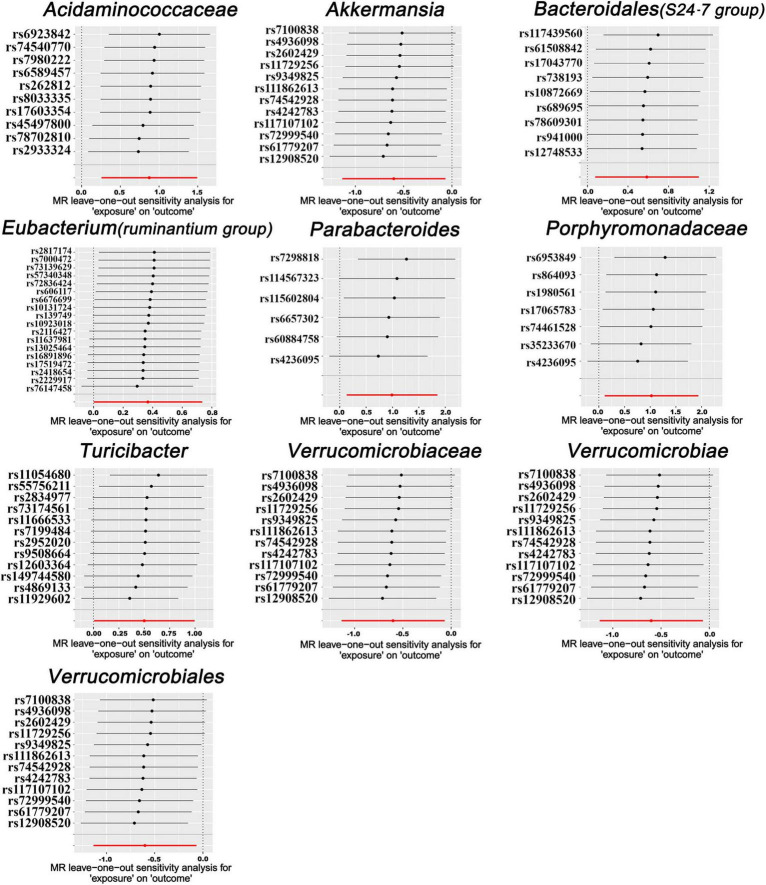
Leave-one-out analysis for MR results. The leave-one-out analysis was conducted by iteratively recalculating MR estimates through the IVW method, systematically omitting one SNP at each iteration. This process aimed to investigate whether any individual SNP with a substantial horizontal pleiotropic effect could significantly impact the MR estimates.

In our forward MR analyses, we employed the IVW method to successfully identified potential causal links between 10 specific bacterial traits and MM risk (Figure 2 and Supplementary Table 4). Notably, we discovered positive relationships between MM risk and the following: family *Acidaminococcaceae* (OR, 2.3947; 95% CI, 1.2937–4.4326; *P* = 0.0054), *Bacteroidales* family S24-7 (OR, 1.7964; 95% CI, 1.0797–2.9890; *P* = 0.0241), family *Porphyromonadaceae* (OR, 2.7848; 95% CI, 1.1246–6.8959; *P* = 0.0268), genus *Eubacterium ruminantium group* (OR, 1.4440; 95% CI, 1.0016–2.0817; *P* = 0.0490), genus *Parabacteroides* (OR, 2.6896; 95% CI, 1.1425–6.3316; *P* = 0.0235), and genus *Turicibacter* (OR, 1.6478; 95% CI, 1.0061–2.6986; *P* = 0.0472) (Figure 2). On the contrary, we found a negative association between MM risk and the following: class *Verrucomicrobia* (OR, 0.5476; 95% CI, 0.3216–0.9322; *P* = 0.0265), family *Verrucomicrobiaceae* (OR, 0.5476; 95% CI, 0.3216–0.9324; *P* = 0.0266), genus *Akkermansia* (OR, 0.5476; 95% CI, 0.3216–0.9324; *P* = 0.0266), and order *Verrucomicrobiales* (OR, 0.5476; 95% CI, 0.3216–0.9322; *P* = 0.0265) (Figure 2). Furthermore, the association between *Bacteroidales* family S24-7 and MM remained consistent when applying the weighted median approach (OR, 2.0346; 95% CI, 1.0714–3.8635; *P* = 0.0300). The scatter plot and funnel plot were displayed Supplementary Figures 1, 2. Comprehensive genetic instruments for evaluating causal effects can be found in Supplementary Table 2. Notably, The lowest F-statistic observed among the instruments stood at 19.5, signifying robust associations between all IVs and the microbiome features. Results from the MR-PRESSO test corroborated our findings (Table 1). Our findings did not suggest the existence of horizontal pleiotropy in the intercept of the MR-Egger regression analysis (Table 1). Additionally, heterogeneity tests showed no noteworthy heterogeneity among these independent variables (Table 1). Importantly, leave-one-out analyses failed to identify any highly influential variants among the instrumental SNPs (Figure 3). In an effort to address potential pleiotropy, we scanned all SNPs used as IVs in our study using the PhenoScanner database. This led to the identification of 8 SNPs associated with secondary traits, and relevant literature was reviewed to explore their connections to MM (Supplementary Table 5). While smoking is associated with various cancers, its link to MM remains unclear (Psaltopoulou et al., 2013). For the other traits in the table, there is currently no substantial evidence linking them to MM. Nevertheless, we conducted MR analyses using the IVW method after excluding these pleiotropic SNPs. Our results continued to support associations between family *Acidaminococcaceae* (OR, 2.2100; 95% CI, 1.1498–4.2479; *P* = 0.0174), *Bacteroidales* family S24-7 (OR, 1.7948; 95% CI, 1.0779–2.9884; *P* = 0.0245), family *Porphyromonadaceae* (OR, 3.71309; 95% CI, 1.2368–11.1471; *P* = 0.01934), and genus *Turicibacter.id.2162* (OR, 1.6475; 95% CI, 1.0041–2.7032; *P* = 0.0481) with MM risk did not change significantly in the IVW method. However, the relationships between family *Verrucomicrobiaceae* (OR, 0.5891; 95% CI, 0.3383–1.0257; *P* = 0.0614), genus *Akkermansia.id.4037* (OR, 0.5892; 95% CI, 0.3384–1.0259; *P* = 0.0615), and order *Verrucomicrobiales* (OR, 0.5891; 95% CI, 0.3383–1.0256; *P* = 0.0614) with MM appeared less stable. These three unstable associations were influenced by a common SNP (rs4936098), but direct evidence linking this SNP’s traits to MM is lacking. Detailed information regarding the results of MR analyses with updated SNPs are provided in Supplementary Table 6.

**TABLE 1:** Effect estimates of the relationships between MM risk and gut microbiotas in the MR analyses.

Gut microbiota	IVW	MR-PRESSO test (IVW)	Pleiotropy Test (IVW)	Heterogeneity Test (MR-Egger)
Class *Verrucomicrobia*	0.0265	0.8560	0.6543718	0.8330287
Order *Verrucomicrobiales*	0.0265	0.8536	0.6543718	0.8330287
Family *Acidaminococcaceae*	0.0054	0.7668	0.9276273	0.7405918
*Bacteroidales* family S24-7	0.0241	0.9683	0.2707392	0.9649069
Family *Porphyromonadaceae*	0.0268	0.5729	0.8650975	0.5399784
Family *Verrucomicrobiaceae*	0.0266	0.8528	0.6532106	0.8328674
Genus *Eubacterium ruminantium group*	0.0490	0.9377	0.6252739	0.9310561
Genus *Akkermansia*	0.0266	0.8515	0.6523758	0.8327713
Genus *Parabacteroides*	0.0235	0.5564	0.5362979	0.4810427
Genus *Turicibacter*	0.0472	0.3613	0.9869001	0.3164009

### 3.2 Backward MR analyses

We further conducted backward MR analyses to investigate potential inverse associations between ten microbial characteristics and MM. Utilizing the IVW method, our analysis failed to reveal any substantiating evidence regarding the similar cause-and-effect relationships between MM and the ten microbial characteristics. We employed three distinct thresholds (SNP *p*-values less than 1.0 × 10^–5^, 5.0 × 10^–6^, or 5.0 × 10^–8^) to choose IVs linked to MM. These three thresholds are commonly used in current MR analysis for SNP selection. Here, we opted for a lower threshold of 1.0 × 10^–5^ and a higher threshold of 5.0 × 10^–8^ for analysis and presentation of results. With a threshold of 5.0 × 10^–8^, only 1 SNP remained, with no statistically meaningful correlations were observed between MM and any of the ten bacterial traits (Table 2). Our results indicate a lack of substantial evidence supporting the notion that MM leads to changes in microbial characteristics using thresholds of 5.0 × 10^–8^ and 1.0 × 10^–5^ (Table 2 and Supplementary Table 7). The findings remained consistent across sensitivity analyses, as outlined in Table 2 and Supplementary Table 7.

**TABLE 2:** Effect estimates of the relationships between MM and gut microbiota were examined in the backward MR analyses.

Gut microbiota	Threshold	Methods	nSNP	OR	95% CI	*p*-value	*p*-value (Intercept)
Class *Verrucomicrobia*	5 × 10^−8^	Wald ratio	1	1.0301	0.9568–1.1091	0.4303	/
Order *Verrucomicrobiales*	5 × 10^−8^	Wald ratio	1	1.0301	0.9568–1.1091	0.4303	
Family *Acidaminococcaceae*	5 × 10^−8^	Wald ratio	1	0.9911	0.9222–1.0652	0.808	/
*Bacteroidales* family S24-7	5 × 10^−8^	Wald ratio	1	0.9667	0.8831–1.0581	0.4623	/
Family *Porphyromonadaceae*	5 × 10^−8^	Wald ratio	1	0.9748	0.9180–1.0351	0.4048	/
Family *Verrucomicrobiaceae*	5 × 10^−8^	Wald ratio	1	1.0301	0.9568–1.1091	0.4303	/
Genus *Eubacterium ruminantium group*	5 × 10^−8^	Wald ratio	1	1.0034	0.9156–1.0995	0.9427	/
Genus *Akkermansia*	5 × 10^−8^	Wald ratio	1	1.0308	0.9574–1.1098	0.4207	/
Genus *Parabacteroides*	5 × 10^−8^	Wald ratio	1	0.9859	0.9273–1.0482	0.6494	/
Genus *Turicibacter*	5 × 10^−8^	Wald ratio	1	1.0162	0.9335–1.1062	0.7104	/
Class *Verrucomicrobia*	1 × 10^−5^	IVW	9	1.0001	0.9672–1.0341	0.9959	0.0741
Order *Verrucomicrobiales*	1 × 10^−5^	IVW	9	1.0001	0.9672–1.0341	0.9959	0.0741
Family *Acidaminococcaceae*	1 × 10^−5^	IVW	9	1.0121	0.9827–1.0424	0.4249	0.3991
*Bacteroidales* family S24-7	1 × 10^−5^	IVW	9	0.9867	0.9510–1.0237	0.4745	0.694
Family *Porphyromonadaceae*	1 × 10^−5^	IVW	9	0.9992	0.9754–1.0235	0.9462	0.3557
Family *Verrucomicrobiaceae*	1 × 10^−5^	IVW	9	1.0001	0.9672–1.0342	0.9932	0.0736
Genus *Eubacterium ruminantium group*	1 × 10^−5^	IVW	9	0.9688	0.9314–1.0078	0.1159	0.4394
Genus *Akkermansia*	1 × 10^−5^	IVW	9	1.0004	0.9675–1.0344	0.9829	0.0742
Genus *Parabacteroides*	1 × 10^−5^	IVW	9	0.9927	0.9686–1.0174	0.5584	0.9469
Genus *Turicibacter*	1 × 10^−5^	IVW	9	1.0194	0.9846–1.0555	0.2778	0.3992

### 3.3 Biological annotation

**FIGURE 4:**
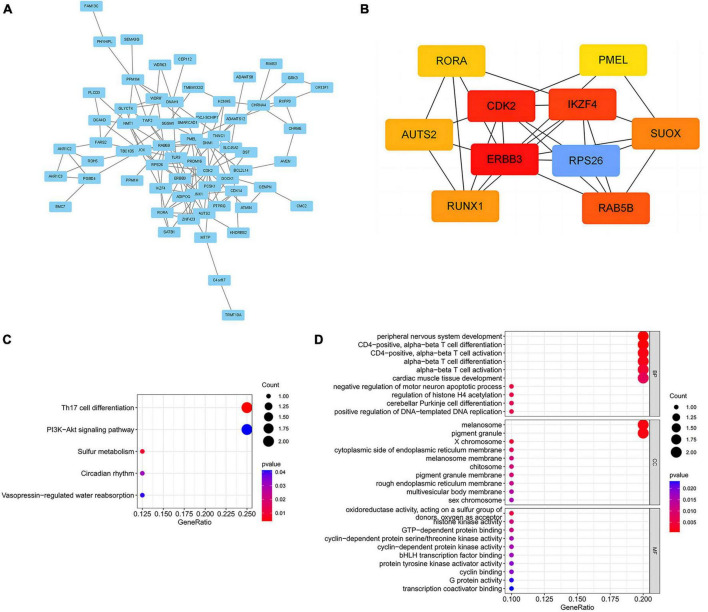
Biological annotation of 74 mapped genes between MM and gut microbial. **(A)** PPI networks of 74 common genes from the STRING database were visualized in Cytoscape, with disconnected nodes omitted for a concise overview. **(B)** The Maximal Clique Centrality (MCC) method implemented in Cytoscape was utilized to ascertain the top 10 hub genes. **(C)** Enrichment analysis of hub genes using the Kyoto Encyclopedia of Genes and Genomes (KEGG) pathways. **(D)** Gene Ontology (GO) pathways analysis of hub genes. BP, biological process; CC, cellular component; MF, molecular function.

**FIGURE 5:**
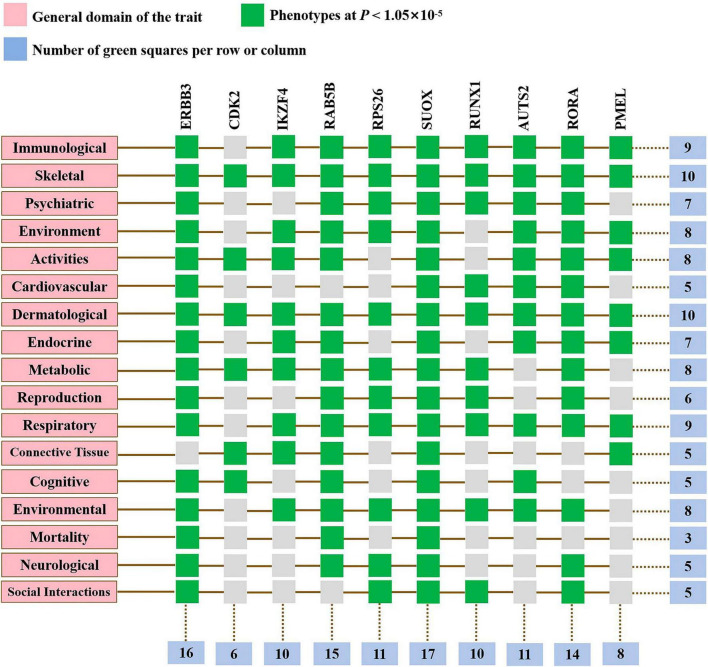
Gene-based phenome-wide association studies. Hub genes displayed heightened genetic signals associated with various domains.

To elucidate the biological mechanisms linking gut microbiota to MM, we annotated the instrumental variables mapping onto 74 genes (Supplementary Table 8). A tightly connected network of 74 shared proteins was revealed through PPI network analysis (Figure 4A). To gain further insights, we used the MCC method to rank the top 10 nodes as hub genes in the PPI network (Figure 4B and Supplementary Table 9). To uncover the possible molecular mechanisms connecting gut microbiota and MM, we performed gene-set enrichment analysis on these hub genes. KEGG pathway enrichment analysis revealed that hub genes were enriched in pathways related to Th17 cell differentiation and sulfur metabolism (Figure 4C and Supplementary Table 10). Additionally, we observed substantial enrichment in processes associated with regulating T cell differentiation and activation through GO analysis (Figure 4D and Supplementary Table 11). Significantly, every hub gene displayed connections with numerous characteristics, even when subjected to the stringent significance threshold of 1.05 × 10^−5^. Gene-based PheWAS revealed that 9 out of 10 hub genes displayed heightened genetic signals associated with immunological domains (Figure 5, Supplementary Figure 3 and Supplementary Table 12).

**FIGURE 6:**
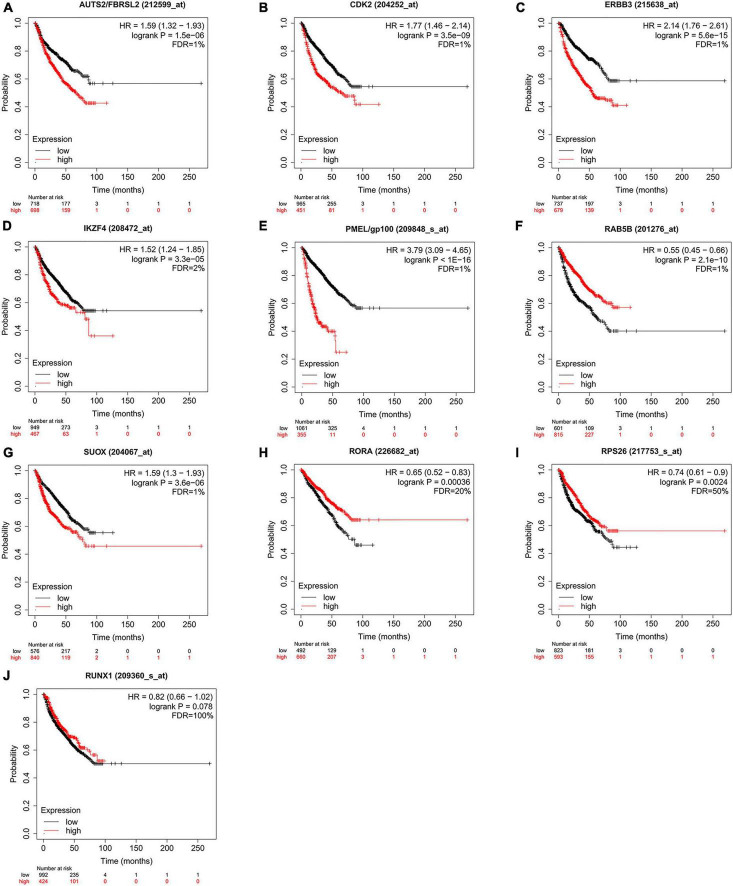
Kaplan Meier survival curves were utilized to evaluate overall survival (OS) among patients diagnosed with multiple myeloma, while also examining the expression levels of hub genes **(A–J)**. Statistically significant findings were determined when all of the following criteria were satisfied: FDR < 5%, HR ≠ 1, *P* < 0.01. FDR, false discovery rate; HR, hazard ratio.

**FIGURE 7:**
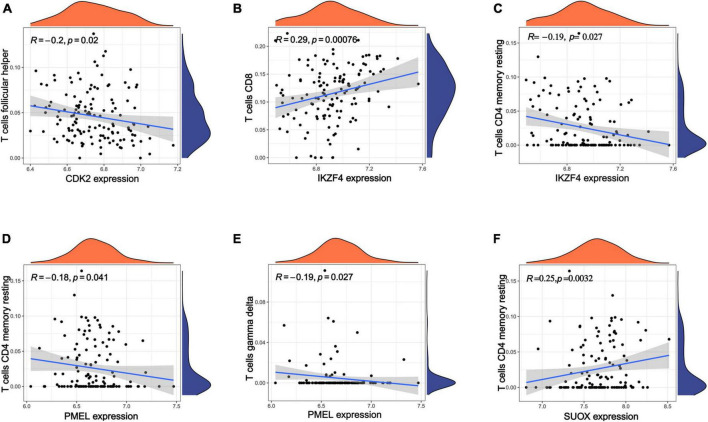
Analysis of immune infiltration in MM using seven hub genes. **(A–F)** Exploring the relationships between hub genes and immune cells. Statistical significance is achieved when *p* < 0.05.

According to the above results, we hypothesize that hub genes exert a substantial influence on the progression of MM. Thus, we utilized the Kaplan-Meier plotter to investigate the association between the expression profiles of hub genes and the overall survival (OS) rates in individuals with MM. We found that higher expression levels of six genes were associated with improved OS in MM patients, whereas high expression of RAB5B was linked to poorer OS (Figure 6). Our GO, KEGG, and PheWAS analyses indicate that hub genes may play a pivotal role in immunological. We leveraged the GSE13591 dataset, which included 133 MM samples and 5 normal donor samples (ND), as a validation set. Gene expression levels underwent normalization through the utilization of the “limma” package within the R software environment. Immune cell infiltration in MM samples vs. normal donors was assessed using the CIBERSORT method, and the results are detailed in Supplementary Table 13. We investigated the correlation between seven hub genes and the abundance of distinct immune cell types within MM samples. The findings were visualized using the “vioplot” packages. Our analysis revealed a notable positive relationship between AUTS2 and activated Mast cells as well as naive B cells, whereas it demonstrated an inverse correlation with resting Mast cells (Supplementary Figure 4A). CDK2 exhibited a positive association with M2 Macrophages and Plasma cells while demonstrating a negative association with T follicular helper cells (Figure 7A and Supplementary Figure 4B). The expression of IKZF4 is positively associated with CD8 T cells and inversely associated with M0 Macrophages and CD4 memory resting T cells (Figures 7B, C and Supplementary Figure 4C). The occurrence of plasma cells exhibited a favorable correlation with PMEL, while it displayed an adverse link with neutrophils, CD4 memory quiescent T cells, memory B cells, and gamma delta T cells (Figures 7D, E and Supplementary Figure 4D). Memory B cells and eosinophils showed a positive correlation with RAB5B, whereas activated mast cells exhibited a negative correlation with RAB5B (Supplementary Figure 4E). SUOX exhibited a positive correlation with memory resting T cells CD4 and Neutrophils, as well as memory B cells, while displaying a negative association with naive B cells and M0 Macrophages in our findings (Figure 7F and Supplementary Figure 4F). These results suggest that these newly identified hub genes are linked to various immune cells in MM, with a particular emphasis on T cells.

## 4 Discussion

Our study conducted two-sample Mendelian randomization analysis and biological annotation by utilizing the existing cost and statistical power of mbGWAS. We obtained the gut microbiota data from MibioGen and the GWAS summary statistics of MM from GWAS Catalog. Next, we conducted a comprehensive investigation into potential causal associations between microbiota features and MM. Finally, we identified novel hub genes that are associated with immunoregulation and clinical prognosis in MM through biological annotation. The results of our forward MR analyses unveiled compelling evidence of both positive and negative causal effects of ten distinct microbial features on MM risk. Specifically, we observed that family *Acidaminococcaceae*, *Bacteroidales* family S24-7, family *Porphyromonadaceae*, genus *Eubacterium ruminantium group*, genus *Parabacteroides*, and genus *Turicibacter* exhibited positive correlations with MM risk. Conversely, class *Verrucomicrobia*, family *Verrucomicrobiaceae*, genus *Akkermansia* and order *Verrucomicrobiales* demonstrated negative correlations with MM risk. While previous studies have highlighted associations between intestinal microbiota and MM, such as *Klebsiella pneumoniae* (Jian et al., 2020), *Clostridium butyricum* (Jian et al., 2020), *Faecalibacterium prausnitzii* (Zhang et al., 2019), *Pseudomonas aeruginosa* (Zhang et al., 2019), and *Prevotella heparinolytica* (Calcinotto et al., 2018). The microbiota revealed in our investigation has not been previously documented in the milieu of MM. *Acidaminococcaceae*, a family in the *Firmicutes* phylum, includes the beer-associated genus *Pectinatus*. Despite being classified as low GC Gram-positive bacteria, *Pectinatus* exhibits unique traits, such as a Gram-negative-like outer membrane and distinctive lipopolysaccharides with remarkable structural heterogeneity (Helander et al., 2004). IL-22 neutralization was found to lead to a reduction in the abundance of the family *Acidaminococcaceae* (Nagao-Kitamoto et al., 2020). Without innate immune defense mechanisms, *Porphyromonadaceae* was discovered to thrive, and it exhibited a higher presence within the male microbiota (Fransen et al., 2017). *Parabacteroides*, a commensal bacterium identified in the gut microbiota, plays a crucial role in mitigating acute pancreatitis, particularly in the context of heparanase-induced exacerbation (Lei et al., 2021). The administration of *Parabacteroides* has been shown to alleviate acute pancreatitis by producing acetate and reducing neutrophil infiltration in both wild-type and heparanase-transgenic mice. *Turicibacter* has shown the ability to predict both immune-related adverse events and the efficacy of immune checkpoint inhibitors (Hamada et al., 2023). *Verrucomicrobia*, *Verrucomicrobiales*, and *Verrucomicrobiaceae* are also related to inflammation (Borton et al., 2017). Liu et al. (2022) found that TLR4 has a substantial influence on RORγt++ regulatory T cell reactions. This interaction with *Akkermansia muciniphila* was shown to exert a noteworthy influence on susceptibility to colon inflammation (Liu et al., 2022). Although our research indicates positive/negative correlations between the aforementioned significant bacteria and MM risk, experimental studies on significant gut microbiota in relation to MM are currently lacking. Therefore, further empirical research is required to substantiate the precise roles and mechanisms of these gut microbiota in the development of MM.

Biological annotation analyses suggest that 74 shared genes are mapped between 10 gut microbiomes and MM. Using the STRING database for PPI network analysis and visualizing it with Cytoscape, we identified top 10 shared genes as hub genes connecting MM and gut microbiota. Enrichment analysis of genes has uncovered connections between hub genes and processes such as T cell differentiation and activation, Th17 cell differentiation, and sulfur metabolism. Additionally, PheWAS analysis revealed a correlation between hub genes and several characteristics, such as metabolic and immunological characteristics, consistent with prior studies (Fan and Pedersen, 2021). Among these hub genes, seven genes (AUTS2, CDK2, ERBB3, IKZF4, PMEL, RAB5B, SUOX) were identified as having prognostic significance in MM patients. AUTS2 demonstrated significantly higher mutation frequencies among African American cases compared to Caucasians. A different investigative study indicates that Hispanic individuals with AUTS2 mutations among MM patients exhibited poorer overall survival (Peres et al., 2022). CDK2 plays a carcinogenic role in MM by participating in multiple signaling pathways, and inhibitors targeting CDK2 have the potential to inhibit the growth of MM (Tu et al., 2011). The ErbB3 atypical expression in MM may have potential effects on tumor cell growth or survival (Walters and Jelinek, 2004). However, the roles of IKZF4, PMEL, RAB5B, and SUOX in the pathogenesis of MM have not been reported yet. T reg cell effector IKZF4 mediates biological processes such as Nr4a factor maintaining T reg cell lineage stability and inhibitory activity (Sekiya et al., 2015). PMEL, also known as gp100, participates in cancer immune infiltration and tumor-specific immunotherapy, especially in targeting T cell therapy (Scheffel et al., 2016). RAB5B, a member of the RAB GTPase family, is pivotal in multiple immune processes, including endocytosis, antigen presentation, and immune signal transduction. SUOX has shown great promise as a diagnostic and prognostic biomarker for various cancers, such as oral squamous cell carcinoma (Nakamura et al., 2018), and hepatocellular carcinoma (Jin et al., 2013). Further bioinformatics analysis demonstrated that six hub genes have a strong connection with different immune cells, particularly T cells, in MM samples. For example, CDK2 exhibited a noteworthy inverse correlation with T follicular helper cells, consistent with previous studies (Niu et al., 2023). IKZF4 exhibited a robust positive correlation with CD8 T cells but displayed a notable inverse relationship with CD4 memory resting T cells. PMEL displayed a significant negative correlation with CD4 memory resting T cells and gamma delta T cells. SUOX exhibited a noteworthy positive association with resting memory CD4 T cells. Therefore, a potential mechanism for the causal link between microbial features and MM may involving the mediation of immune system changes by hub genes, particularly in the context of T cell differentiation and activation.

Despite these valuable insights, it is crucial to recognize several constraints inherent to the current study. To begin with, the examination of bacterial taxa was restricted to the genus category, with no investigation conducted at finer levels like species or strain. Next, although the majority of participants in this GWAS hail from European backgrounds, it’s worth noting that the inclusion of a limited number of individuals from diverse ethnicities could potentially impact the outcomes. Consequently, it is important to recognize that our findings may have certain constraints when applied to different racial demographics. Individuals of European ancestry constitute approximately 78% of the total population, while other ancestries make up the remaining 22%. Thus, a noteworthy limitation arises from the disparity in ancestral backgrounds between the data in MiBioGen, which may pose a potential threat to the independence assumption due to population stratification. We chose IVs associated with gut microbiota at a threshold of *p* < 1.0 × 10^−5^, which exceeds the conventional genome-wide significance level (*p* < 5 × 10^−8^). This higher threshold was necessary to ensure we had a sufficient number of IVs for our MR analyses, as only one IV remained when selecting IVs at the genome-wide significance level (*p* < 5 × 10^−8^). In the future, we anticipate achieving more dependable outcomes with MR by significantly expanding the sample size while adhering to stricter criteria. While we have pinpointed several possible causal connections in our MR analyses, it is crucial to emphasize that these findings demand additional confirmation. In our pursuit of a more robust understanding, not only should we contemplate the expansion of our sample size, but we must also direct our attention toward the advancement of novel techniques aimed at bolstering the statistical potency of mbGWAS. Furthermore, it is imperative that we embark on further investigations into the biological mechanisms at play, with a specific focus on elucidating the central genes that act as pivotal links in the intricate interplay between the host genome of patients with MM and their gut microbiome.

In summary, our study has identified several gut microbiota that may possess the potential to impact the risk of MM. We also identified novel host-microbiome shared genes linked to immune regulation and clinical prognosis in MM. These results could hold considerable clinical implications for MM prevention and therapy. Nevertheless, further investigations are needed to elucidate the specific mechanisms that could serve as potential intervention targets for MM.

## Data availability statement

The data presented in this study are available at public database, accession link: human multiple myeloma GWAS (data available at: http://ftp.ebi.ac.uk/pub/databases/gwas/summary_statistics/GCST 90042001-GCST90043000/GCST90042760/); human gut microbiome GWAS (data available at: https://mibiogen.gcc.rug.nl); Gene Expression Omnibus (data available at: https://www.ncbi. nlm.nih.gov/geo/); Kaplan-Meier plotter database (data available at: http://kmplot.com/analysis/index.php?p=service&cancer); STRING database (data available at: https://string-db.org/).

## Author contributions

ZF: Conceptualization, Methodology, Supervision, Writing – original draft, Writing – review and editing. ML: Conceptualization, Writing – review and editing. JB: Data curation, Methodology, Writing – review and editing. YL: Data curation, Methodology, Writing – review and editing. YC: Investigation, Supervision, Writing – review and editing. LiZ: Methodology, Supervision, Validation, Writing – review and editing. XG: Investigation, Visualization, Writing – review and editing. LL: Conceptualization, Funding acquisition, Supervision, Validation, Writing – original draft, Writing – review and editing. LSZ: Conceptualization, Funding acquisition, Supervision, Validation, Writing – original draft, Writing – review and editing.

## Footnotes

1
http://ftp.ebi.ac.uk/pub/databases/gwas/summary_statistics/GCST90042001-GCST90043000/GCST90042760/

2
https://mibiogen.gcc.rug.nl

3
https://www.ncbi.nlm.nih.gov/geo/

4
http://kmplot.com/analysis/index.php?p=service&cancer

5
https://string-db.org/
