# Supplementary material for: Exploring the causal relationship between gut microbiota and multiple myeloma risk based on Mendelian randomization and biological annotation
Source: Front Microbiol. 2024 Feb 12;15:1310444. doi: 10.3389/fmicb.2024.1310444 (PMC10895040; doi:10.3389/fmicb.2024.1310444)
Supplement: Supplementary file 1 [file Data_Sheet_1.PDF]

frontiers

Supplementary Material

Figure S1

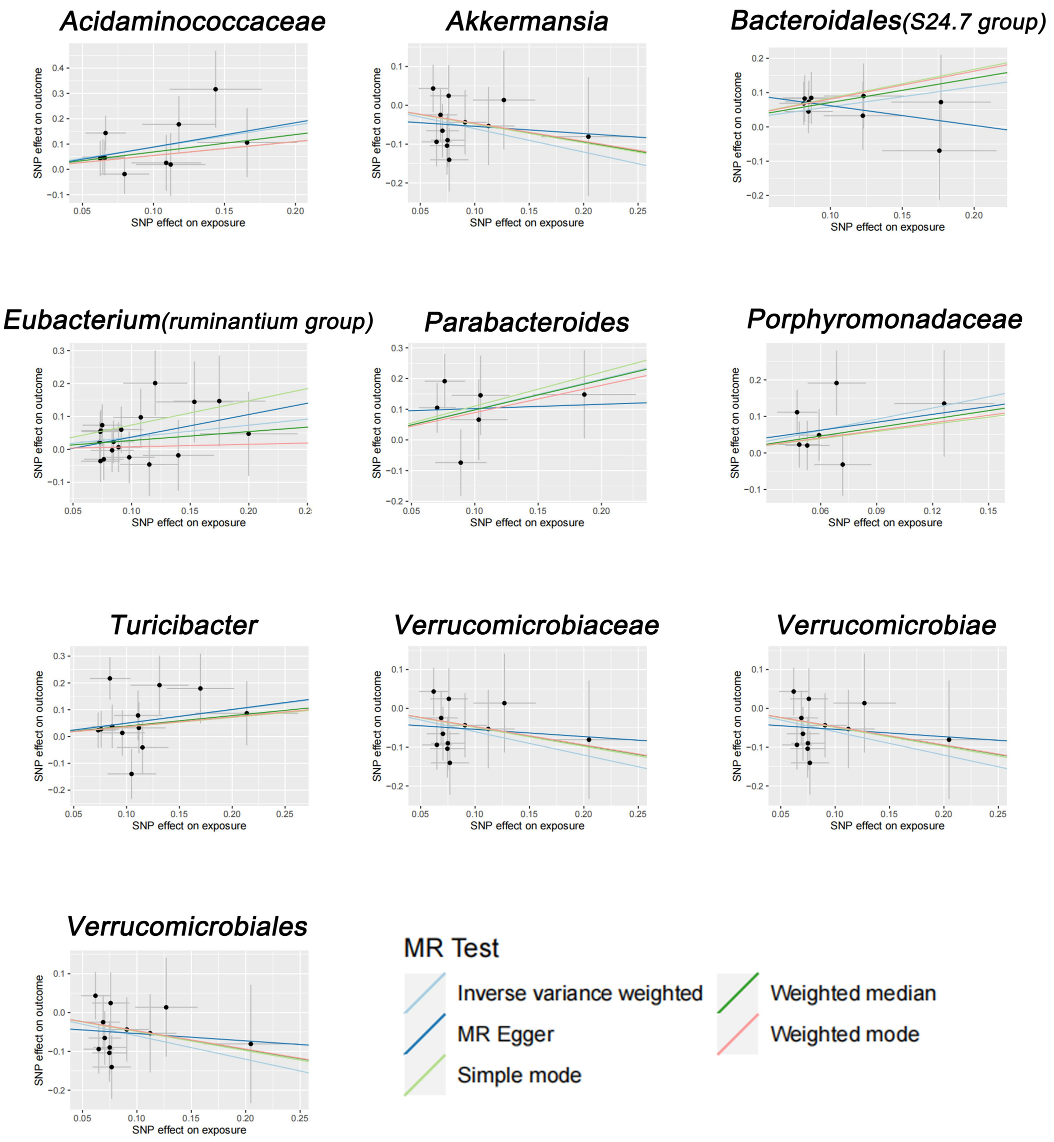

Figure S1. Scatter plot from the forward MR results. Scatter plot illustrates the relationships between the effects of single nucleotide polymorphisms (SNPs) on the gut microbiota and their effects on multiple myeloma.

Figure S2

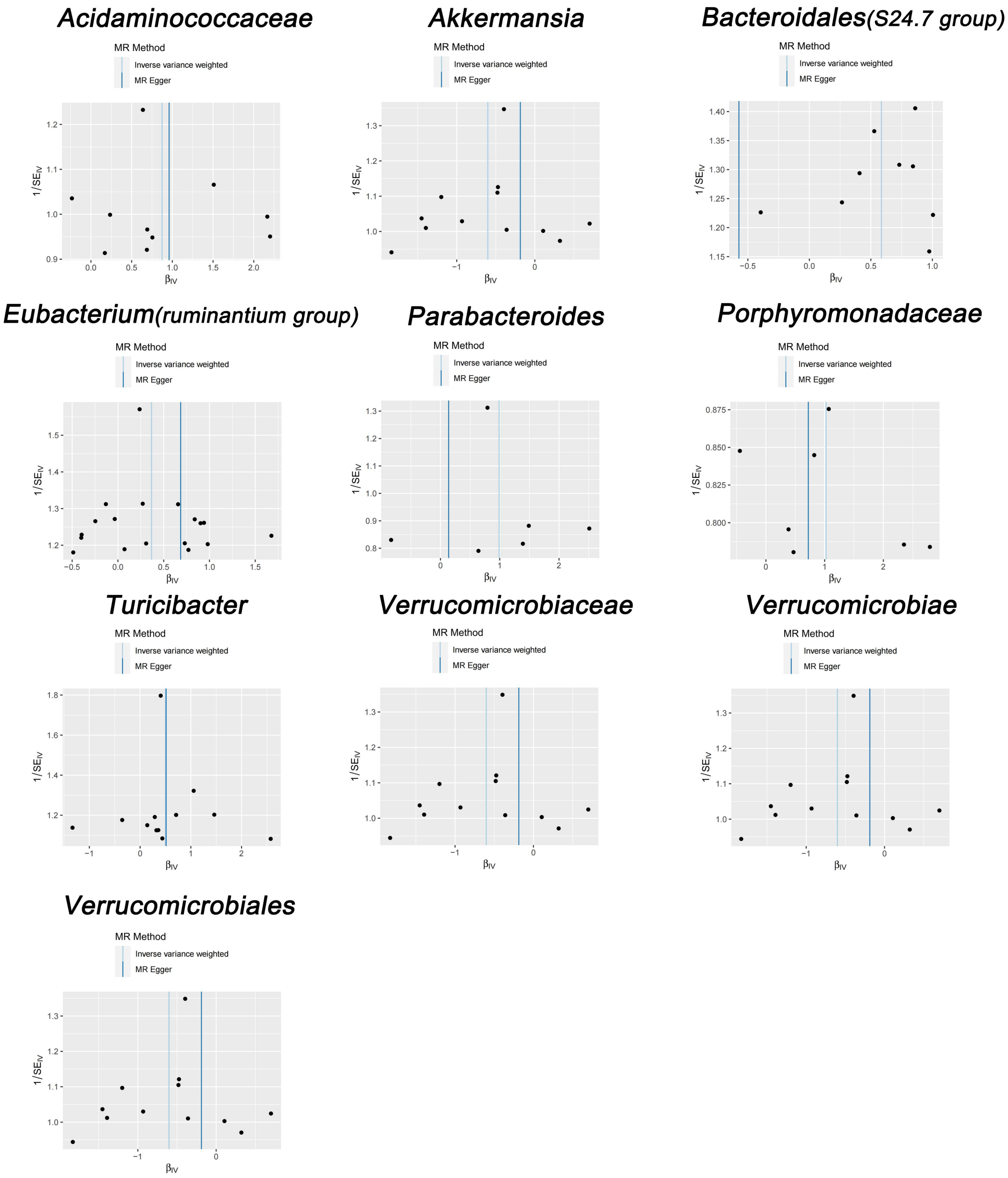

Figure S2. The funnel plot illustrating the causal connection between gut microbiota and multiple myeloma risk is displayed, with individual single nucleotide polymorphisms depicted in the background.

Figure S3

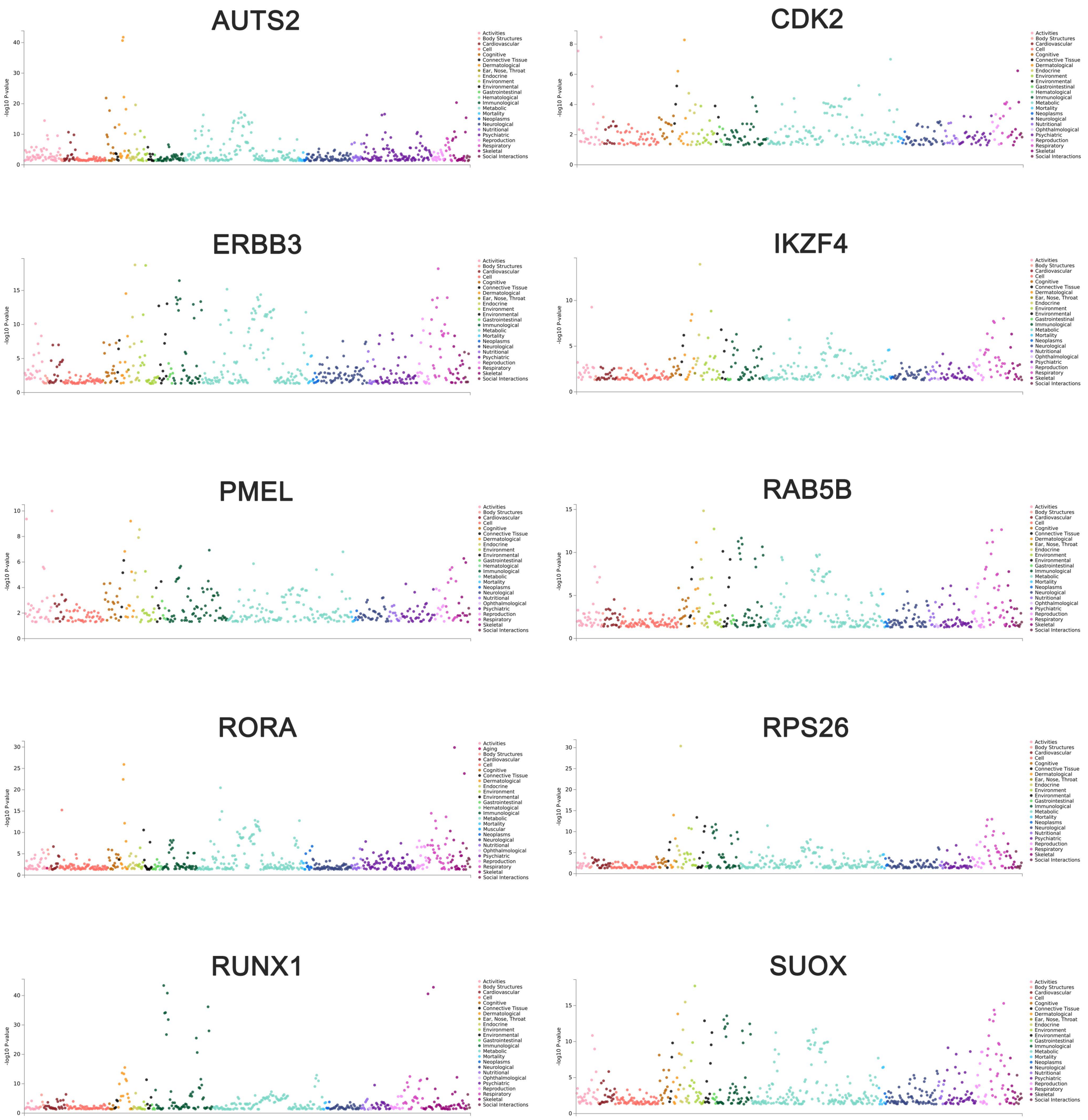

Figure S3. PheWAS analyses with GWAS ATLAS. Traits ordered by domain, overall number of GWASs considered for these analyses: 4,756. Default p-value cutoff at 0.05 and Bonferroni corrected $p = 1.05{\times}10^{-5}$.

Figure S4

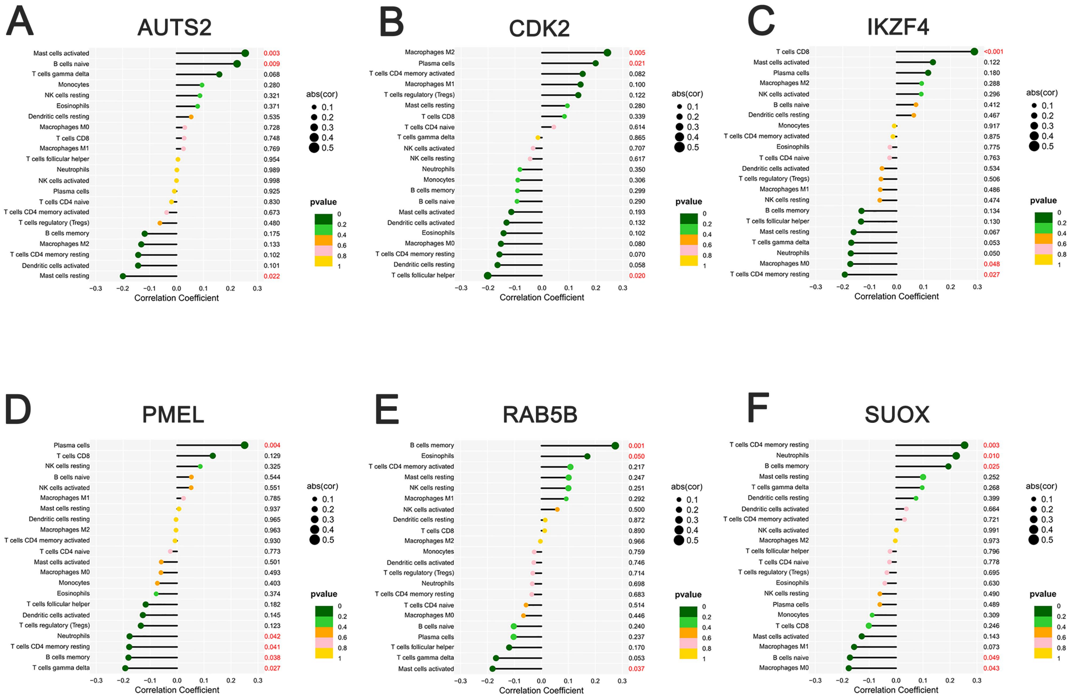

Figure S4. Analysis of immune infiltration in MM using seven hub genes. (A-F) Exploring the relationships between seven hub genes and infiltrating immune cells. The results are considered statistically significant when $p < 0.05$.
